# Volatile Profiles of Emissions from Different Activities Analyzed Using Canister Samplers and Gas Chromatography-Mass Spectrometry (GC/MS) Analysis: A Case Study

**DOI:** 10.3390/ijerph14020195

**Published:** 2017-02-15

**Authors:** Santino Orecchio, Michele Fiore, Salvatore Barreca, Gabriele Vara

**Affiliations:** 1 Dipartimento di Scienze e Tecnologie Biologiche, Chimiche e Farmaceutiche. Università di Palermo, Viale delle Scienze, 90128 Palermo, Italy; 2 Agenzia Regionale per la Protezione dell’Ambiente della Sicilia, Corso Calatafimi 219, 90129 Palermo, Italy; mfiore@arpa.sicilia.it (M.F.); gabrielevara@libero.it (G.V.); 3 Agenzia Regionale per la Protezione dell’Ambiente della Lombardia, 20100 Milano, Italy; salvatore.barreca@unipa.it; 4 Euro-Mediterranean Institute of Science and Technology (Iemest), 90128 Palermo, Italy

**Keywords:** canister, indoor, volatile organic compounds (VOCs), Palermo

## Abstract

The objective of present study was to identify volatile organic compounds (VOCs) emitted from several sources (fuels, traffic, landfills, coffee roasting, a street-food laboratory, building work, indoor use of incense and candles, a dental laboratory, etc.) located in Palermo (Italy) by using canister autosamplers and gas chromatography-mass spectrometry (GC-MS) technique. In this study, 181 VOCs were monitored. In the atmosphere of Palermo city, propane, butane, isopentane, methyl pentane, hexane, benzene, toluene, meta- and para-xylene, 1,2,4 trimethyl benzene, 1,3,5 trimethyl benzene, ethylbenzene, 4 ethyl toluene and heptane were identified and quantified in all sampling sites.

## 1. Introduction

Due to industrialization and expansion of the urban periphery, the distance between residential areas and productive ones (crafts, industrial, commercial) has been gradually reduced. In this context, volatile compounds (often with a bad smell) produced by gas emissions from several activities, have been implicated as a cause of life quality decrease for neighboring communities and, in some cases, result in negative consequences for human health and welfare [1,2,3]. For this reason, volatile emissions (especially odorous ones) are becoming an important problem, particularly with respect to complaints that cause poor relationships between residents and craftsmen, manufacturers and environmental control authorities.

The gaseous emissions generally consist of a large number of volatile organic compounds (VOCs) in varying mixing ratios [4,5,6]. The updated U.S. Environmental Protection Agency (EPA) [7] definition concerning VOCs is as follows: “VOCs are organic chemical compounds whose composition makes it possible for them to evaporate under normal indoor atmospheric conditions of temperature and pressure” (USEPA, 2011) [8]. The National Research Council [9] described VOCs as “organic compounds that vaporize easily at room temperature”.

Volatile compounds (often consisting of hazardous chemicals) can be transported to areas very far from their sources of emission, thereby increasing the risk to which human populations are exposed. Like other aerial pollutants, VOCs can also affect health at concentrations above thresholds. The key symptoms associated with exposure to some VOCs that have undesirable effects for humans and animals include conjunctive irritation, nose and throat discomfort, headache, allergic skin reaction, nausea, emesis, epistaxis, fatigue and dizziness [3]. Exposure to some VOCs during normal indoor activities in different occupations has been linked to cancer risks [2,3].

The health risks associated with volatile compounds detected in indoor and outdoor samples from landfills and other common activities have not yet been assessed. As is well known, chronic health effects (non-cancerous or cancerous) of VOCs could be a result of inhalation and long-term exposure to polluted air [4].

In literature, little information is available regarding the composition of volatile emissions from activities commonly located within towns, especially in Mediterranean cities like Palermo (Italy). For example, during the processing of municipal solid waste in landfills, VOCs are generated as intermediate or end products [10,11]. The methods for their emission characterization and quantification generally are of three kinds [12,13,14,15]:

Analytical (chemical analyses);
Sensorial: (dynamic olfactometry);
Senso-instrumental: (electronic nose)

Analytical methods allow determination of the composition of gas mixtures using several separation and identification techniques, for example gas chromatography-mass spectrometry (GC-MS). This technique is repeatable and accurate but it is difficult to relate the chemical composition of an odorous emission to its olfactory properties. Another downside, especially when using detectors that do not give specific information about the molecules (FID (Flame Ionization Detector), ECD (Electron Capture Detector), etc.), is the difficulty of identifying an established and limited number of compounds, especially when present at low concentrations.

Sensorial methods, such as dynamic olfactometry, use the noses of a group of qualified examiners as sensors but there are several factors that may influence odor perception. The most important one is the subjectivity and variability of human senses.

An electronic nose detects and discriminates among complex volatile mixtures using a sensor array. A compound generates a characteristic fingerprint that could be used to construct a database so that unknown odors can subsequently be classified and identified [16]. In this context, it is important to identify, as in our case, the volatile compounds constituting the emission of a particular activity by using gas chromatography technique.

Several techniques for sampling the constituents of composting volatile emissions are being developed (absorption on solid materials followed by thermal desorption, cold trap adsorption or solvent extraction, and enrichment through several cold traps or through reaction reagents) [17,18,19,20]. Some authors used different methods, for example, a three-grade cold trap combined with GC-MS, dynamic olfactometry and electronic noses, to evaluate emissions from a landfill site located in northern Italy [15].

The objective of present study was to identify volatile organic compounds emitted as a result of several activities being undertaken in Palermo by using canister autosamplers and GC-MS technique. In addition, quantitative determinations were carried out at several stations. The overall aim was to obtain the profiles of volatile compounds from sources within or near the city (landfills, motor vehicle traffic, fuel distributors, construction works, etc.). Generally, source profiles are composition patterns of species emitted from a source category. In the present study, composition patterns are expressed (as an example) as the chromatographic peak area fraction of each compound relative to the total area of all the compounds in the source emission. Source profiles or signatures are used to identify major pollution sources. The VOC pattern and their changes can be used as a tool for identification of sources, sinks and transport processes [21]. 

In Italy, sample collection by canister is not common practice. In contrast, this type of sampler is used in the U.S. in several air monitoring programs and constitutes the basis for the Environmental Protection Agency’s Compendium Method TO-15 [22]. The stability of several volatile compounds and their halogenated derivatives in canisters is well-known [23,24]. The sampling of air in electropolished canisters, followed by pre-concentration with a cryofocusing step (used by us), is a simple, flexible, reliable, sensitive and interference-free method for VOCs as per the USEPA (Washington, DC, USA) [25]. In addition, it is advantageous because it is possible to carry out several analyses on the air same sample. The preparation and storage of standard mixtures in canisters ensures the accuracy and precision during the instrumental calibration.

In the present work, the qualitative profiles of 181 volatile compounds (see supplementary material) resulting from different activities and sources in thirty sites located in Palermo city zone were acquired. In particular, several analyses were carried out on air sampled at a municipal solid waste landfill that collects the waste from the city of Palermo and other neighboring municipalities. Also, the concentrations of 64 volatile compounds at stations located in the city of Palermo (urban traffic) were quantified.

## 2. Materials and Methods

This paper presents the results of several sampling campaigns carried out in the urban and periphery of Palermo (Table 1) using canister autosamplers. The campaigns were carried out over six months (July 2013–January 2014) and the samples were collected in non-rainy days.

### 2.1. Chemicals

A standard gaseous VOC mixture containing 64 compounds (Scotty analyzed gases, *p* = 1300 psig, accuracy ±5%, V = 0.75 L, from 0.96 to 1.05 ppmV) and internal standard mixture containing four compounds (bromochloromethane, 1.4-difluorobenzene, chlorobenzene d_5_, 1.4-bromofluorobenzene) (10 ppmV; Scotty analyzed gases, *p* = 1800 psig, accuracy ±5%, V = 0.75 L) were purchased from Air Liquide America Specialty Gasses LLD Sott-Restekand (Sigma Aldrich, Saint Louis, MO, USA).

### 2.2. Quality Assurance

The uncertainty of the entire analytical process (sampling and analyses) was preliminarily tested by applying the scheme proposed by UNI (Ente nazionale italiano di unificazione) [26] and Eurachem [27,28]. To this aim, in 10 different stations (n° 1–10; see underlined stations in Table 1), air sampling was performed in duplicate, using two identical canisters. For all canisters, the sampling time (7 min) was carefully established in laboratory by using calibrated flow restrictors. To evaluate the uncertainty of the analytical process (Table 2), the VOC content in samples was quantified relative to the internal standard added to the sample, according to the EPA TO-15:1999 method [29].

### 2.3. Site and Stations Description

Samples were collected from Palermo (Italy). Palermo is a city (about 850,000 inhabitants) with a heavy load of vehicular traffic and major industrial and craft activities occur within the city area. It is characterized by conspicuous air pollution [30,31,32]. The town is situated on the northwestern coast of the island along the wide bay Piana di Palermo and is overlooked by a mount (Mt. Pellegrino, 600 m above sea level). It is delimited on the northeast by the Tyrrhenian Sea and it is surrounded by mountains 500–1000 m above sea level.

The characteristics and geographic coordinates of the sampling stations are reported in Table 1. Locations were chosen to maximize capture of the volatile plume from different activities. The 35 sampling stations were distributed as follows:

- Twelve air samples were collected in the Palermo urban area (samples n° 1–10, n° 13a,b), in particular, samples n° 13a and n° 13b were collected from a petrol station;
- Two samples (n° 11 and n° 12) were collected directly from the emissions of the fuel tanks of two cars, powered by gasoline and diesel, respectively;
- Two samples (n° 11a and n° 12a) were taken from the previous cars from the exhaust pipes;
- Two samples (n° 14, n° 15) were taken at the Bellolampo Municipal solid waste landfills that collect the garbage from the city of Palermo and other neighboring municipalities. In particular, sample n° 15 was taken in the vicinity of a large tank that collects the leachate. The Bellolampo landfill, one of the largest landfills in Sicily, is part of the municipality of Palermo, north-west of the city center. The site, which is between 364 m above sea level (the lower part of the reclaimed historic landfill) and 536 m above sea level, converges to the east with a valley and is bordered to the north and south-east by heights. The nearest inhabited settlement is located about 1 km south of the area;
- Four air samples (from n° 16 to n° 19) were collected at different points in the area around the landfill, within a radius of about 2 km;
- One air sample was collected during a fire in a waste dumpster located in the previous area (n° 20);
- One sample was obtained during a fire in a depot of waste plastics (n° 21);
- During different production activities (n° 22 citrus processing, n° 23 hairdresser, n° 24 dental, n° 25 stone and marble processing, n° 26a,b coffee roasting, n° 27a,b fried food cooking, n° 28 wood painting) nine air samples were obtained;
- Two samples (n° 29 and n° 30) were taken inside two homes during use of incense and scented candles. In the past, candles have been utilized as a source of light and during the day are frequently used, together with incense, for decorative and religious purposes in indoor environments. Candles and incense burning produce smoke during the long, slow, and incomplete combustion process. The smoke emitted by these objects has been proven to contain hazardous substances [19,33,34,35] and has also been identified as mutagenic using the Ames test [35].

The vacuum canisters used in this study for sampling air are made from specially modified stainless steel containing chromium and are passivated, using, e.g., the Summa technology.

### 2.4. Canister/GC-Analysis

This steel treatment ensures analyte stability, which generally depends on the type of canister, the means of scrubbing, the reactivity of the material and the conditions of storage [36,37]. Before air sampling, canisters were cleaned with nitrogen 5.0 and tested using gas chromatography (GC-MS). Before sampling, the canisters were evacuated to 0.060 mmHg. Air samples were collected in a vacuum canister by means of free flow. We carried out an instantaneous sampling by calibrating the valve in the laboratory to sample in 7 min. The blank concentrations were checked and were close to the detection limit for the considered compounds. Air samples were collected using stainless steel canisters, V = 6000 mL, in the urban and peripheral area of Palermo.

The canister samples were returned to the laboratory and introduced to the GC-MS by mean cold and trap dehydration technique (CTD) to preconcentrate samples. The instrument used for analyses was a GC system model 450 (Varian Inc., Palo Alto, CA, USA) equipped with a cryogenic pre-concentration system 7100A (Entech Instruments Inc., Simi Valley, CA, USA). Air samples were loaded and pre-concentrated directly by an automated cryofocusing system using liquid nitrogen, according to the EPA methodology [29]. Injection volume was 200–1000 mL, according to the practical concentrations. The pre-concentrated gas samples then were injected to GC-MS.

The qualitative analysis of volatile compounds was performed by an HP 6890 gas chromatography system coupled with the 5973 mass spectrometer detector 5973 (GC-MS, Agilent Technologies, Santa Clara, CA, USA) with an Entech 7100 Preconcentrator. A DB-624 (6% cyanopropylphenyl-94%-dimethylpolysiloxane) capillary column (60 m × 0.32 mm × 1.80 μm, Aglient Technology, Santa Clara, CA, USA) was used with helium 5.5 as carrier gas at a rate of 3 mL·min^−1^. The injector and detector temperature were 100 and 240 °C, respectively. The GC oven temperature was programmed at 32 °C, held for 10 min, increased to 150 °C at a rate of 5 °C·min^−1^, then increased to 230 °C at 15 °C·min^−1^ and held for 6 min. The mass detector was run in full scan mode with m/z = 35–300. The scanning method allows for qualitative information to be obtained through research in the libraries (Wiley 275) of the instrument.

VOC identification in the standard mixture was carried out by comparing the spectra of the single components with those stored in the library acquisition system under the same experimental conditions. VOC identification in the sample was carried out on the basis of previously determined retention times and confirmed using mass spectra. The most abundant ion was used for quantification, while other ions were additionally used for the confirmation. 

Standard gas mixtures were employed for concentration calibration and quality control assurance. The calibration gases were prepared by a pressure dilution method, dynamically diluting the standard mixture containing 64 compounds to 1, 2, 3, 4, 6, 8 and 10 ppbV (from 1 × 10^−7^% to 1 × 10^−6^% *v*/*v*), with pure nitrogen and the same concentration (10 ppbV) of internal standards (bromochloromethane, 1.4-difluorobenzene, chlorobenzene d_5_, 1.4-bromofluorobenzene) used for all samples. The method of dynamic dilution for gases is a recent issue (ISO 6145-1 2003; ISO 7 2009) and there are some dilution systems already available based on different principles and configurations.

We used a dynamic dilution system that employs a mass flow meter that avoids the disadvantages of the traditional method, reducing to just one or two the certified reference materials needed for each quantity. All VOC calibrations had good dose–response correlations (r^2^ = 0.9911–0.9999) within the concentration range investigated. The single volatile compound content in the sample was quantified with respect to the internal standard added to the sample.

In addition to the quantitative analyzes performed in stations 1–10, profiles of the volatile substances present in the neighborhood of specific activities were recorded. In these cases, a total of 181 volatile compounds were monitored. The identification of analytes in the unknown samples was carried out on the basis of 80% similarity of their mass spectra compared with those of the instrument library. 

## 3. Results

The uncertainties of the analytical process (sampling and measures) have been reported in Table 2 for the nine considered analytes (ranging from 25% to 42%) that well-matched with the scope of environmental monitoring. Once assessed the applicability of the quantitative method to air sampled by the canisters, the concentrations of 64 compounds (Table 3) at stations located in the city of Palermo were determined. In order to discuss and compare the sample compositions, we used internal normalization procedures. The results of the qualitative analysis carried out on the gas mixtures sampled by the canister near the activities or sources are reported in Table 4.

### 3.1. Fuels

Very few of profiles reported in literature include abundances much beyond elemental carbon, organic carbon, metals, and the relatively few organic compounds known to be markers of various classes of motor vehicle emissions. Most are not representative of traffic emissions. In this study, two source profiles were established for fuels, including petrol and diesel fuel; in addition, two profiles for emission from petrol and diesel fuel (after combustion) were established to evaluate differences between them. There are significant differences in chemical compositions among different fuels. Pentane, 2-methylpentene, 2-methyl-2-butene, hexane, 3-methylhexane, benzene, toluene and m,p-xylene are the major species of volatile organic compounds in gasoline (emissions sampled directly in the tank) and in the emissions (sampled from exhaust pipe) from vehicles powered by this fuel. Table 4 shows the chemical species determined in diesel fuel sampled directly in the tank and in emissions from diesel engines sampled from the exhaust pipe. In the first case, the top compounds were butane, isopentane, hexane, benzene, heptane, methyl cyclohexane, toluene, octane, m,p-xylene, nonane, decane and 1,2,4-trimethylbenzene, while in the engine fuel emissions (Table 4), only cyclopentene, hexane, nitromethane, benzene, toluene and m,p-xylene were detected.

### 3.2. Urban Area (Motor Vehicle Traffic)

Several volatile organic compounds were quantified in the urban atmosphere of Palermo (Table 3). Among the most abundant VOCs, butane, isopentane, 2-methylpentane, hexane, benzene, toluene, ethylbenzene, m,p-xylene and 1,2,4-trimethylbenzene were identified in all sampling sites. In all sites, the qualitative distribution of VOCs is similar. The parameters that, during the entire monitoring, provided values higher than the respective quantification limits are shown in Table 3. Only for the 4-ethyltoluene and the 1,3,5-trimethylbenzene, in some cases, were the results lower than the LOQ (Limit of quantification). Most of the target analytes detectable in the air of zones with high vehicular traffic coincide with those produced by the combustion of fuels (gasoline and diesel). The highest concentrations of VOCs were detected at station n° 7 (Leonardo da Vinci) during all the sampling surveys (four). Leonardo da Vinci road is placed in a commercial and busy area, in which more pollutants are produced from vehicular emissions.

In addition, the Re Ruggero, Basile and Indipendenza 2 sites (stations n° 3, 4, 8) are adjacent to city center, surrounded by a popular resort area and many commercial shops, where total and single VOCs levels are also appeared to be significant.

Benzene/toluene (B/T) ratios in ranged from 0.18 to 0.42 and are in good agreement with data in the literature. In detail, ratios measured in research carried out in urban busy areas ranged from 0.15 to 0.35, indicating the presence of higher amounts of toluene than benzene in this type of emission compared to other activities. A B/T ratio of 0.5 has been used by several authors [36,37,38,39] as an indicator of traffic emissions. On another hand, urban air measurements have also showed B/T ratios ranging between 0.27 and 0.5 [38]. Landfills have appreciably lower benzene/toluene ratios (i.e., 0.1 in USA) [36]. A wide range of B/T ratios has been observed in the downtown of Los Angeles [39,40] and Mexico City [41]. The benzene/toluene ratio is mainly dependent on fuel composition, which is variable from company to company, country to country, and season to season [42]. On the other hand, some authors [42,43] used a B/T ratio of approximately 0.5 to characterize vehicular emissions and higher ratios have been found from combustion of bio-fuel, charcoal and coal. This suggests that strong toluene sources come from not only traffic but also other emissions, such as gasoline vapor, paint, and industrial activities involving the use of toluene, which makes its special distribution complex. One sampling (station n° 13) was performed near fuel distribution. Butane, acetone, dichloromethane, hexane, benzene, toluene, m,p-xylene, 1,2,4-trimethylbenzene and ethylbenzene were identified. In these cases, it is interesting to highlight that the vehicular traffic has a more considerable impact on gas emissions than the direct fuel emissions. The results obtained in this research paper are in agreement with results of our previous studies that measured other environmental contaminants [30,31,32,44].

### 3.3. Municipal Solid Landfill

Landfill volatile compounds are generated by the decomposition of organic material contained in wastes. The intermediate and products of waste in degradation processes generate many types of volatile gases [4,5,6,12].

The objective of profile acquisition in the Bellolampo landfill (stations n° 14 and n° 15) was to establish the volatiles compounds produced by decomposition of urban waste and to evaluate their diffusion in the surrounding area. The samplings were carried out each month during the period July 2013–January 2014 (except October) within the landfill (stations n° 14 and n° 15) and in four external points (stations n° 16–19).

The main volatile compounds in the municipal solid landfill emissions (Table 4) were ethanol, cyclopentene, hexane and limonene, while in close proximity to the leachate tank area, acetone, dichloromethane, hexane, toluene, limonene and p-cymene were detected. These data are in good agreement with previous results of some authors, affirming that limonene is a typical tracer of fresh waste, and p-cymene is the characteristic compound of leachate and biogas [6]. Alcohols are representative compounds of fresh waste [45]. According to several authors [46,47], alcohols and carboxylic acids are intermediates of waste decomposition and contribute appreciably to landfill emissions. In good agreement to literature data, Bellolampo landfills have an appreciably higher toluene concentration than benzene (qualitative); benzene/toluene ratios measured in other researches [37,39] ranged from 0.015 to 0.11 indicating the presence of higher amounts of toluene than benzene in landfills compared to traffic exhaust rich urban areas. 

### 3.4. Suburban Areas External to the Landfill

Table 4 shows the compounds detected in four remote stations external to the landfill (stations n° 16–19). We can hypothesize that the abundant presence of hexane and dichloromethane directly depends on the landfill. This transport is justified by the performance of winds that were monitored during the sampling period. In addition, in the station n° 16 (SP1 (Provincial Street, Km 7) isopentane, pentane, acetone, nitromethane, toluene and m,p-xylene were detected. These compounds are attributable by vehicle emissions. This source is justified considering that the monitored area is adjacent to the road travelled by the garbage truck continuously. This conclusion is confirmed by comparison profiles of station n° 16 and those of combusted fuels and urban area ones, described previously. The widespread substances in station n° 17 (Contrada Fimmina Morta) are ethanol, acetone, dichloromethane, hexane, and, in August, limonene was also detected. We think that in this area, the presence of hexane and dichloromethane depends on the landfill, due to the high presence of the considered compounds in the same period in stations n° 14 and n° 15. This transport is justified by considering wind direction. In addition to compounds that generally characterized landfills, we found 1,3,5-trioxane.

This compound, in the monitored area, was produced by combustion of plastic materials; in fact, residues of fire were detected during the sampling.

From the volatile substances (Table 4) sampled in the station n° 18 (Contrada Inserra) it can be observed that, in addition to the characteristic compounds of the landfill (ethanol, acetone, dichloromethane, hexane, etc.), considerable amounts of toluene and p-cymene were detected only during one sampling (5 December 2013). These substances, as already mentioned, can be attributable to the landfill leachate. In fact, in the same period, abundant rains caused a widespread leakage of leachate from the tank localized in the area opposite to station n° 18. 

**Figure 1:**
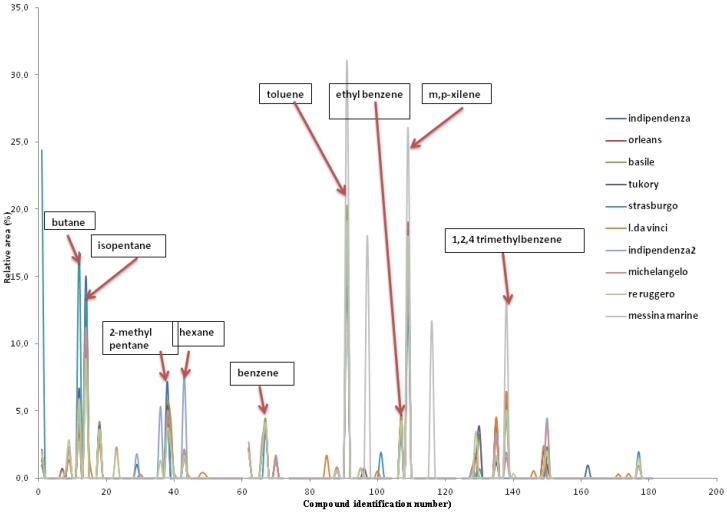
Profiles of urban traffic.

The fourth sampling, in the surrounding landfill area, was carried out in the balcony of an apartment located on Via Piazza Armerina (station n° 19). In this station, some components are those that characterize landfill emissions. In detail, limonene was detected only in August, November and December. Also, in this case, toluene, m,p-xylene and 1,2,4-trimethylbenzene were found. The latter was found in the emissions of urban areas (Figure 1) and was derived from vehicular car traffic. The profile of this monitoring site in August is slightly different from that of previous surveys. In this case, benzene, toluene, styrene, benzaldehyde, limonene, indene, naphthalene and undecene were detected. This apparent anomaly can be ascribed to fire of a waste bin, which took place in the same area. The profile recorded directly on the garbage bin (not reported) during the fire was very complex, however, benzene, toluene, styrene, benzaldehyde, limonene, indene, undecene and cyclooctatetraene (cyclic hydrocarbon due to pyrogenic sources) profiles were determined.

One advantage of current analytical approaches is that by applying temperature-dependent pyrolysis GC-MS, researchers [47] have identified a wide range of VOCs from municipal solid waste (MSW), including plastics.

It is also interesting to note that, among the volatile substances produced during a fire of plastic waste (station n° 20), benzene, toluene, styrene, propane, 2-methylfuran, decene and ethylbenzene were found.

Limonene has also been detected in the station n° 22 located near a citrus products processing area (lemons, oranges, mandarins, etc.) to produce concentrate juices and essences. However, considering that in the citrus products industry other substances in addition to limonene such as αβ-pinene and γ-terpinene were found, it is possible differentiate the emission produced by the processing of citrus fruits with that of the landfill.

### 3.5. Coffee Roasting

Some air sampling using canisters were carried out near coffee roasters, which are located in the inner city. During the roasting phase, prevalent substances such as propane and acetone were detected. Moreover, in the cooling phase performed using an air current, acetone, benzene, toluene, m,p-xylene and styrene are released from hot coffee. The presence of the propane can be attributed to the incomplete combustion of the liquid gas used to produce the heat necessary to toast coffee.

### 3.6. Street Food Laboratory

Urbanization processes, are often associated with increase in ready-to-eat foods. Generally, street food stalls are placed in most congested streets, areas with high traffic, close to manufacturing activities, bus/train stations, etc. In Italy and in particular in Palermo, street foods provide a wide range of products and nutrients, helping people to meet their dietary needs. Large availability, assortment and low cost make street foods an affordable option. Considering the large number of activities that prepare food ready to eat in Palermo, we performed some analysis in a laboratory that prepares fried foods (potatoes, fish, timbale of rice, etc.). 

Contrary to our expectations, in the indoor sampled air, we found ethanol, toluene and p-xylene and traces of acrolein. Only the latter can be attributed to the specific activity, while we can assume that characteristics compounds of vehicular emissions car, may come from the neighboring street because the laboratory is equipped with an efficient air exchange system, taking “clean” air from outdoor environment that is contaminated by urban traffic emissions. 

### 3.7. Building Work

To study VOC profiles (not reported) produced in home renovation processes [48], indoor samples during a home renovation were collected. The house was located in the countryside, in an area with low density of vehicular traffic (cars). Sampling was conducted after use of products used in wood protection (impregnating). Monitoring results show the presence of several hydrocarbons (heptane, octane, ethylbenzene, m,p-xylene, nonane, decane, etc.). In this case, hydrocarbons are not attributed to vehicular traffic because several compounds such as hexane, benzene, toluene and 1,2,4 trimethylbenzene, that generally were products of vehicular emission, were not detected. Therefore, we can assume that contaminants found are used in commercial impregnating as solvents.

### 3.8. Use Indoor of Incense and Scented Candles

Indoor air quality (IAQ) has been an important issue in indoor pollution for more than half a century. It is known that IAQ deterioration depends on large number of chemicals, or classes of chemicals, detectable in indoor air [2,18,19,20,49,50,51]. 

Candles and incense, since ancient times, have been utilized as a source of light and for religious purposes, today, in indoor environments, they are frequently used for decorative, religious and emotional purposes. Candles and incense burning produce smoke during a long, slow, and incomplete combustion process.

The smoke emitted by wax has been proven to contain several hazardous compounds (aldehydes, phenol, aromatic and aliphatic hydrocarbons, PAHs (Polycyclic Aromatic Hydrocarbons), etc.) and has also been identified as mutagenic [33,34,35]. 

During use of incense and scented candles, two analyses in indoor environment were performed. During the incense burning benzene, acetaldehyde, ethanol, styrene and toluene were found, while during the scented candle burning ethanol, and at low concentrations, limonene and acetaldehyde were found. 

### 3.9. Dental Laboratory

In the air of a dental laboratory, we identified methyl methacrylate and traces of propane. The first compound is a component of the resin used for dental fillings. 

### 3.10. Processing of Stones and Marbles

During the processing of stones and marbles, a similar situation to that of the dental laboratory was found. In particular, during the resin coating of the surfaces, toluene and styrene were emitted. These chemicals come from the resin used during the finishing of surfaces that is composed of 32%–36% styrene, while, during the drying of the artifacts, in an oven fueled with liquid gas propane was emitted, probably due to incomplete combustion.

## 4. Principal Component Analysis

**Figure 2:**
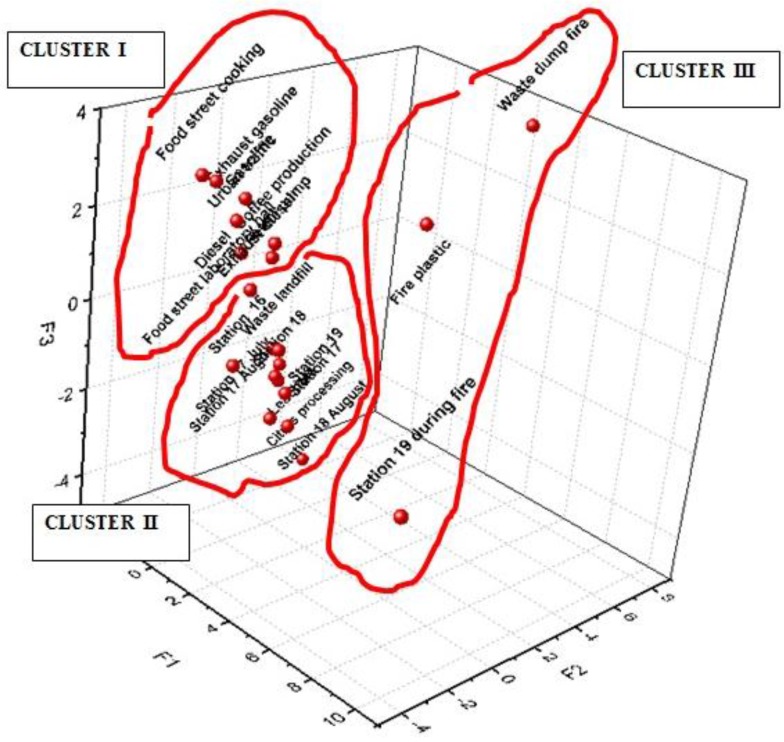
Principal component analysis.

Principal component analysis (PCA) as the multivariate analytical tool is normally used to reduce a set of original variables and to extract a small number of latent factors (principal components, PCs) in order to analyze relationships among the observed variables. As result of an effective ordination process, the first PC accounts for the greatest proportion of the original variance, while the second as well as the following PCs progressively explain smaller data variations. In this study, PCA analysis was carried out in order to point out the possible effect of different volatile organic compounds in sampling sites. The variance of 43% was explained by three eigenvectors–principal components. Although the total variance of system is low, it is possible to identify three different clusters. In detail, the individuated clusters are related at different sampling site positions at different periods (near landfills, near vehicular emissions and during fires) (Figure 2). The first principal component (PC1) is able to differentiate two different clusters. In detail, the fist cluster with a PC1 value greater than one determines the sampling sites from during the fire, while the second cluster has PC1 value lower than that of other samples. The second PC (PC2) explains the sampling site with the plastic and waste-pump fire, while the third principal component (PC3) is able to differentiate sampling sites with landfill contamination and vehicular contamination (gasoline).

## 5. Conclusions

In this study, sampling using canisters and analysis by gas chromatography with mass spectrometric detector has been validated. Using the validated method, we found most of the volatile substances present in emissions from several sources (fuels, landfill leachate, etc.); these were produced by activities that characterize the productive economy and the lifestyle of a European city, in particular that of Palermo. 

Data obtained by GC-MS analysis of volatile compounds sampled using canisters in the vicinity of a landfill or close to other activities can be used, using suitable algorithms as references for the calibration and for interpretation of data collected by electronic nose and the subsequent compound quantification. 

The results of this study indicate that it is possible to differentiate the main volatile compounds emitted during municipal solid waste treatment and other activities using GC-MS and canisters. In particular, taking into account that the volatile compounds produced by vehicles and other activities are different from those generated by the decomposition of waste, the profiles registered in areas far from the landfill permit differentiation of the volatile compounds produced by the decomposition of waste and transported by the winds from those commonly produced from vehicular traffic. In addition, canisters similar to those used in this study could be installed onboard drones to sample in areas particularly difficult to access.

## Figures and Tables

**Table 1:** Sampling stations (The underlined stations are those for which quantitative data are reported).

N°	Emission	Description	Coordinates
1	Urban traffic	Indipendenza Square	38°06′38.67″ N–13°21′04.30″ E
2	Urban traffic	Messina Marine St.	38°06′54.63″ N–13°22′33.50″ E
3	Urban traffic	Re Ruggero St.	38°06′31.96″ N–13°21′14.39″ E
4	Urban traffic	Basile St.	38°05′57.57″ N–13°20′45.58″ E
5	Urban traffic	Tukory St.	38°06′30.95″ N–13°21′30.90″ E
6	Urban traffic	Strasburgo St.	38°09′24.75″ N–13°19′34.87″ E
7	Urban traffic	Leonardo da Vinci Av	38°07′36.27″ N–13°19′14.90″ E
8	Urban traffic	Indipendenza Square 2	38°06′38.67″ N–13°21′04.30″ E
9	Urban traffic	Michelangelo St	38°07′49.18″ N–13°18′18.88″ E
10	Urban traffic	Orleans Park	38°06′35.09″ N–13°21′30.90″ E
11	Fuel	Gasoline	38°06′52.22″ N–13°20′16.87″ E
11a	Fuel combustion	Gasoline combustion	38°06′52.22″ N–13°20′16.87″ E
12	Fuel	Diesel fuel	38°06′52.22″ N–13°20′16.87″ E
12a	Fuel combustion	Diesel fuel combustion	38°06′52.22″ N–13°20′16.87″ E
13	Fuel distributor	Fuel distributor	38°04′56.83″ N–13°25′55.05″ E
14	Waste emission	Landfill waste (Bellolampo)	38°08′36.13″ N–13°16′17.09″ E
15	Waste emission	Landfill waste (leachate)	38°08′36.13″ N–13°16′17.09″ E
16	Waste emission	Area adjacent to the landfill (7 km)	38°08′13.38″ N–13°16′19.94″ E
17	Waste emission	Area adjacent to a landfill	38°08′06.46″ N–13°14′49.15″ E
18	Waste emission	Area adjacent to a landfill	38°08′58.05″ N–13°17′52.87″ E
19	Waste emission	Armerina street	38°07′47.65″ N–13°17′43.66″ E
20	Waste emission	Waste dumpster fire	38°07′47.65″ N– 13°17′43.66″ E
21	Waste emission	Fire during plastic recycling	37°58′32.04″ N–13°42′38.09″ E
22	Industrial emission	Citrus processing	38°05′22.45″ N–13°24′03.50″ E
23	Professional emission	Hairdresser	38°07′10.38″ N–13°21′05.82″ E
24	Professional emission	Dental laboratory	38°07′07.12″ N–13°21′14.84″ E
25	Professional emission	Laboratory (stone and marble)	38°09′58.89″ N–13°18′23.62″ E
25a	Professional emission	Laboratory (stone and marble)	38°09′58.89″ N–13°18′23.62″ E
26	Professional emission	Coffee roasting	38°10′00.30″ N–13°18′19.99″ E
27a	Professional emission	Street food (hall)	38°06′29.21″ N–13°20′45.34″ E
27b	Professional emission	Street food (cooking)	38°06′29.21″ N–13°20′45.34″ E
28	Professional emission	Wood painting	38°02′59.40″ N–13°29′43.34″ E
29	Indoor activity	Burning incense	38°05′37.37″ N–13°24′05.08″ E
30	Indoor activity	Burning scented candles	38°06′52.22″ N–13°20′16.87″ E

**Table 2:** Uncertainty of the analytical process.

Compound	RSD% Sampling	RSD% Analysis	RSD% Process	U% Process (k = 2)
Benzene	7.2	10	12	25
Heptane	10	14	17	34
Toluene	7.1	12	14	28
Ethylbenzene	9.3	14	17	34
p-Xylene, m-Xylene	12	15	19	39
o-Xylene	12	15	20	40
4-Ethyltoluene	14	14	20	40
1,3,5 Trimethylbenzene	15	14	20	40
1,2,4-Trimethylbenzene	17	12	21	41

The average concentrations (169 ppbV = 1.69 × 10^−5^% *v*/*v*) were about 6 times of that at the Orleans site (station n° 10). This site lies in a cultural and recreational area, there are scattered university buildings and a large green area. RSD = Relative Standard Deviation, U = Uncertain.

**Table 3:** Concentration in ppbV of principal compounds in air samples.

Station	Benzene	Heptane	Toluene	Etyl Benzene	m,p-Xylene	o-Xylene	4 Ethyl Toluene	1,3,5 Trimethyl Benzene	1,2,4 Trimethyl Benzene
1	3.4	0.98	12	2.2	9.1	3.1	2.1	0.94	3.6
1	3.7	1.0	12	2.2	9.3	3.2	2.2	0.95	3.8
1	3.4	0.95	11	2.1	8.7	3.0	2.1	0.93	3.6
1	3.5	1.0	12	2.2	9.0	3.2	2.1	0.96	3.6
2	1.0	1.2	5.9	1.1	3.7	1.2	<LOQ	<LOQ	1.0
2	1.1	1.1	6.1	1.1	3.8	1.2	<LOQ	<LOQ	0.97
2	1.1	1.2	6.2	1.1	3.9	1.2	<LOQ	<LOQ	1.2
2	1.1	1.2	6.4	1.2	4.1	1.2	<LOQ	<LOQ	1.1
3	6.1	2.0	20	3.8	16	6.0	5.2	2.6	11
3	5.6	2.0	19	3.7	15	5.8	4.8	2.5	9.9
3	5.7	1.9	19	3.9	15	6.5	5.3	2.8	12
3	5.6	1.8	19	3.7	14	5.9	4.2	2.1	8.2
4	3.4	1.0	11	1.9	7.7	3.2	2.0	0.65	4.0
4	3.4	0.95	11	1.9	7.8	3.1	2.0	0.59	3.9
4	3.2	0.98	11	1.9	7.7	3.2	2.0	0.60	4.1
4	3.2	0.92	11	1.8	7.2	3.2	1.9	0.50	3.6
5	6.3	2.3	23	4.3	16	6.3	1.9	2.4	9.9
5	6.3	2.3	23	4.3	16	6.2	1.9	2.3	9.7
5	5.9	2.1	22	4.0	16	6.2	2.0	2.4	10
5	6.1	2.2	22	4.1	16	6.2	1.9	2.3	9.6
6	2.1	0.46	5.8	0.95	3.9	1.4	0.45	<LOQ	0.78
6	2.1	0.46	5.8	0.94	3.8	1.3	0.40	<LOQ	0.70
6	2.0	0.46	5.5	0.94	3.7	1.3	0.45	<LOQ	0.85
6	2.0	0.47	5.6	0.96	3.8	1.3	0.61	<LOQ	0.76
7	15	4.6	46	9.3	32	13	11	5.2	21
7	15	4.6	45	9.1	32	12	11	5.2	21
7	16	5.1	50	10	36	14	12	5.9	24
7	16	5.6	52	11	41	16	14	7.1	30
8	7.1	2.2	24	3.0	12	4.3	3.5	1.2	4.7
8	7.2	2.2	24	3.0	12	4.4	3.3	1.2	4.6
8	7.8	2.3	25	3.3	13	4.7	3.8	1.3	5.2
8	7.6	2.2	24	3.2	12	4.6	3.8	1.3	5.1
9	3.6	1.3	9.3	1.5	6.7	2.2	1.3	0.50	2.2
9	3.5	1.2	8.9	1.4	6.3	2.1	1.2	0.43	1.9
9	3.5	1.2	9.2	1.5	6.5	2.1	1.4	0.48	2.1
9	3.5	1.2	9.2	1.5	6.6	2.2	1.5	0.51	1.9
10	4.2	1.2	10	1.8	7.7	2.7	1.7	0.57	2.1
10	4.1	1.1	10	1.8	7.4	2.5	1.7	0.58	2.1
10	3.9	0.96	9.2	1.6	7.1	2.4	1.7	0.62	2.2
10	4.1	1.0	9.7	1.7	7.3	2.4	1.8	0.61	2.3

LOQ = Limit of quantification.

**Table 4:** Profiles of different activities (see complete a volatile organic compound list in Supplementary Materials, Figure S1).

Compound/Activity	Gasoline	Exhaust Gasoline	Diesel	Exhaust Diesel	Urban Traffic	Petrol Pump	Waste Landfill	Leachate	Station 16	Station 17	Station 18	Station 19	Station 19 During Fire	Waste Dump Fire	Fire Plastic	Citrus Processing	Coffee Production	Food Street Laboratory	Food Street Laboratory
1,2,4-trimethylbenzene			x		x	x												x	
2 methyl propene																	x		
2-methyl-2-butene	x	x																	
2-methylfuran														x					
2-methylpentane	x	x			x														
2-methylpentene																			
3-methylhexane																			
acetone						x		x	x		x						x		
acroleine																		x	
benzaldehyde													x	x					
benzene	x	x	x	x	x	x						x	x	x	x		x		
butane					x	x								x				x	
cyclooctatetraene																			
cyclopentene			x	x			x												
decane																			
decene														x	x				
dichloromethane						x		x	x	x	x	x	x						
ethanol										x	x	x	x					x	
ethylbenzene					x	x								x	x		x		
furan														x	x				
heptane																			
hexane			x	x	x	x	x	x	x	x	x	x	x		x				
indene													x						
isopentane			x	x	x				x			x	x						
limonene										x				x					
m,p-xylene	x	x	x	x	x	x	x	x	x			x	x				x	x	
methyl cyclohexane																			
Methyl furan													x		x				
methyl methacrylate													x						
naphthalene																			
nitromethane				x					x										
nonane																			
octane																			
p-cymene								x			x					x			
pentane	x	x																	
propane															x		x	x	
styrene													x		x				
toluene				x	x	x			x	x	x	x	x	x	x		x	x	
undecene													x						
α pinene																x			
β-pinene																x			
γ-terpinene																x			

X = Analyte present in the sample.

## Supplementary Materials

The following are available online at www.mdpi.com/1660-4601/14/2/195/s1, Figure S1: VOCs investigated.
