# Supplementary material for: Volatile Profiles of Emissions from Different Activities Analyzed Using Canister Samplers and Gas Chromatography-Mass Spectrometry (GC/MS) Analysis: A Case Study
Source: Int J Environ Res Public Health. 2017 Feb 15;14(2):195. doi: 10.3390/ijerph14020195 (PMC5334749; doi:10.3390/ijerph14020195)
Supplement: Supplementary file 1 [file ijerph-14-00195-s001.pdf]

Supplementary Materials: Volatile Profiles of Emissions from Different Activities Analyzed Using Canister Samplers and Gas Chromatography-Mass Spectrometry (GC/MS) Analysis: A Case Study

Santino Orecchio, Michele Fiore, Salvatore Barreca and Gabriele Vara

id	Compound	id	Compound	id	Compound	id	Compound
1	1-Propene	47	1-Propanol	93	Butyl acetate	139	α-Methylstyrene
2	Propane	48	2-Butene, 2,3-dimethyl	94	1-Octene	140	2-Methylstyrene
3	1-Propyne	49	3-Methylcyclopentene	95	Octane	141	Benzaldehyde
4	Dichlorodifluoromethane	50	2-Methylfuran	96	2-Acetylfuran	142	3-Methylstyrene
5	1,2-Propadiene	51	Butanal	97	Hexamethylcyclotrisiloxane	143	δ-3-Carene
6	Methyl chloride	52	2,3-Butanedione	98	Spiro[2,4]Hepta-4,6-Diene	144	α-Terpinene
7	1-Butene	53	Nitromethane	99	Butyl acetate	145	Benzofuran
8	1,3-Butadiene	54	2-Butanone	100	N-ethyl-1,3-dithioisoindoline	146	Limonene
9	1-Propene- 2 methyl /isobutene	55	3-Buten-2-one	101	Tetrachloroethylene	147	4-Methylstyrene
10	1-Butyne	56	3-Methyl-1,3-pentadiene	102	2-Hexanone	148	p-Cymene
11	Acetaldehyde	57	(Z) 2-Methyl-1,3-pentadiene	103	Hexanal	149	1,3,5-Trimethylbenzene
12	Butane	58	Methylcyclopentadiene	104	2,4-Dimethyl-1-heptene	150	1,2,3-Trimethylbenzene
13	Methylcyclopropane	59	2,4-Dimethyl 1,4-pentadiene	105	Chlorobenzene-D5	151	Benzene, 3-butenyl
14	Isopentane	60	Ethyl Acetate	106	1,3-Cyclooctadiene	152	Butylbenzene
15	Ethylcyclopropane	61	Bromochloromethane	107	Ethylbenzene	153	3-Methyldecane
16	Methane, fluorotrichloro	62	2-Methylhexane	108	1-Nonene	154	γ-Terpinene
17	1-Pentene	63	Cyclohexane	109	m,p- Xylene	155	1,2-Diethylbenzene
18	Pentane	64	Carbon Tetrachloride	110	Phenylacetylene	156	Undecane
19	1,2-Butadiene	65	2-Methyl-1-propanol	111	Nonane	157	Benzyl chloride
20	Ethanol	66	3-Methylhexane	112	Styrene	158	1,2-Dichlorobenzene
21	Cyclobutane, methylene	67	Benzene	113	1,3,5,7-Cyclooctatetraene	159	1-Undecene
22	Furan	68	Cyclohexene	114	2,6-Dimethyloctane	160	1-Propynylbenzene
23	2-Methyl-1-butene	69	1-Heptene	115	α-Thujene	161	o-Isopropenyltoluene
24	Ethyl Chloride	70	Heptane	116	o-Xylene	162	2,5-Dimethylstyrene
25	2-Methyl-2-butene	71	2-Butenal	117	α-Phellandrene	163	2-Methylbenzaldehyde
26	2-Propenal	72	(Z)-2-butenal	118	α-Pinene	164	Phenylacetaldehyde
27	Propanal	73	1,4-Difluorobenzene	119	Heptanal	165	1-Methyl-2-(2-propenyl)benzene
28	1,1,2-Trichloroethane	74	1,2-Dichloropropane	120	Cumene	166	2-Methylbenzofuran
29	Acetone	75	2-Heptene	121	2-Butoxyethanol	167	7-Methylbenzofuran
30	Carbon disulfide	76	ethyl propionate	122	4-Methylnonane	168	m-Cymene
31	2-Propanol	77	1,4-Hexadiene, 4-methyl	123	2-Methylnonane	169	3-Methylindole
32	(Z)-1,3-Pentadiene	78	3-Methyl-3-buten-2-one	124	m-Bromofluorobenzene	170	1-Dodecene
33	Cyclopentadiene	79	2-Ethylfuran	125	2-Methyl-2-cyclopentenone	171	Indene
34	Methyl acetate	80	4,4-Dimethyl-cyclopentene	126	3-Methylnonane	172	1-Methylindene
35	Cyclopentene	81	2,5-Dimethylfuran	127	Camphene	173	Terpinolene
36	Dichloromethane	82	1,3,5-Trioxane	128	Propyl benzene	174	1,2-Dimethyl-4-ethylbenzene
37	Cyclopentane	83	1-Butanol	129	1-Methyl-4-ethylbenzene	175	(E)-decahydronaphthalene
38	2-Methylpentane	84	Methylcyclohexane	130	1-Ethyl-3-methylbenzene	176	1,3,5-Trichlorobenzene
39	3-Methylpentane	85	Methacrylic acid	131	Propenylbenzene	177	Hexachloro-1,3-butadiene
40	1-Butene, 2,3-dimethyl	86	Dimethyl disulfide	132	Decane	178	1,1a,6,6a-tetrahydrocyclopropa[a]indene
41	Acrylonitrile	87	Iso butylacetate	133	Myrcene	179	1,2-Dihydronaphthalene
42	1-Hexene	88	4-Methylheptane	134	β-Pinene	180	Azulene
43	Hexane	89	2-Butenenitrile	135	1-Ethyl-2-methylbenzene	181	Naphthalene
44	2-Hexene	90	4-Methylcyclohexene	136	1-Decene		
45	2,5-Dihydrofuran	91	Toluene	137	2,6-Dimethylnonane		
46	2-Methylbutanal	92	2-Butyl acetate	138	1,2,4-Trimethylbenzene		

Figure S1. Investigated compounds.
